# Pediatric orthopaedic lower extremity trauma and venous thromboembolism

**DOI:** 10.1007/s11832-015-0697-1

**Published:** 2015-10-12

**Authors:** Robert F. Murphy, Manahil Naqvi, Patricia E. Miller, Lanna Feldman, Benjamin J. Shore

**Affiliations:** Department of Orthopaedic Surgery, Boston Children’s Hospital, 300 Longwood Ave, Hunnewell 221, Boston, MA 02115 USA; Department of Orthopaedics, Medical University of South Carolina, 96 Jonathan Lucas Street, Charleston, South Carolina 29425 USA

**Keywords:** Venous thromboembolism, Pediatric orthopaedics, Trauma, Lower extremity, Fracture

## Abstract

**Purpose:**

Research on venous thromboembolism events (VTE), such as deep venous thrombosis (DVT) and pulmonary embolism (PE), in pediatric orthopaedic trauma patients is sparse. We describe the incidence in the USA of VTE associated with pediatric lower extremity orthopaedic trauma, and characterize injury patterns and VTE treatment methods.

**Methods:**

The Pediatric Health Information System (PHIS) was queried from 2004 to 2013 using ICD-9 codes for lower extremity fractures (pelvis, femur, tibia, ankle, foot) and dislocations (hip, knee, ankle, subtalar) and VTE. Records were queried for age, diagnoses, and VTE treatment.

**Results:**

During the study period 285,611 clinical encounters reported lower extremity trauma. Of those, 167 patients were simultaneously coded with VTE (99 DVT, 50 PE, 18 combined DVT/PE), to give an incidence of VTE associated with pediatric lower extremity trauma of 0.058 %. Patients were from 39 centers, with an average age of 12.9 years (range 0–19). There were 249 fractures and 21 dislocations, with 25 (15 %) patients sustaining more than one lower extremity injury. The most common fracture locations were the femur/femoral neck (95), tibia/ankle (92), and pelvis (44). 72 % (121/167) of patients were treated with anticoagulation medication, of which the most common was low-molecular-weight heparin (111/167, 66 %).

**Conclusions:**

The incidence of VTE events associated with lower extremity orthopaedic trauma is 0.058 %. Adolescents and polytrauma patients with injuries of the femur/femoral neck, tibia/ankle, and pelvis are more commonly affected. Low-molecular-weight heparin is commonly used to treat VTE in pediatric and adolescent patients.

## Introduction

Venous thromboembolism events (VTE) include deep venous thrombosis (DVT) and pulmonary embolism (PE). In adults, orthopaedic procedures and traumatic injuries are known risk factors for VTE [1–3], while traumatic injuries, infections, and central venous catheters have also been linked to VTE in pediatric patients [4, 5].

Current literature regarding VTE in children with orthopaedic trauma is limited by small cohorts of patients [6] or survey responses [7, 8]. Recently, data on VTE associated with elective orthopaedic procedures has been published [9], but there is no national data in the USA on VTE events associated with pediatric orthopaedic trauma.

The primary purpose of this study was to report the incidence of VTE associated with pediatric orthopedic lower extremity trauma. We also reviewed the injury locations and treatment methods for VTE in this patient population.

## Methods

Following Institutional Review Board approval, the Pediatric Health Information System (PHIS) was queried from 2004 to 2013. PHIS is an administrative database that contains inpatient, emergency department, ambulatory surgery, and observation encounter-level data from over 45 pediatric hospitals in the United States. Records are de-identified at the time of data submission, and data are subjected to a number of reliability and validity checks before being included in the database. We searched for all patient encounters with simultaneous coding of lower extremity orthopaedic trauma and a lower extremity DVT and/or a pulmonary embolus. (See Table 1 for ICD-9 codes).

**Table 1:** ICD-9 codes used to query the PHIS database for VTE events and lower extremity trauma

DVT	453.40, 453.41, 453.42
PE	415.1
Pelvic fracture	808.*
Femur fracture	821.* and 820.*
Tibia fracture	823.* and 824.*
Ankle fracture	824.*
Foot fracture	825.*, 826.*
Hip dislocation	835.*
Knee dislocation	836.*
Ankle dislocation	837.*
Subtalar dislocation	838.*

## Results

From 2004 to 2013, there were 36,524,804 unique encounters submitted to the PHIS database, of which 285,611 contained a diagnosis of orthopaedic lower extremity trauma (0.7 %). Of these cases, 167 qualified for inclusion with a concomitant diagnosis of VTE during the same encounter. This represents an incidence of 0.00046 % associated with all clinical encounters, and an incidence of 0.058 % (95 % confidence interval = 0.050–0.068 %) specifically associated with orthopaedic lower extremity trauma. The average age at diagnosis was 12.9 years (range 0–19). The cohort of patients came from 39 different institutions across the United States.

**Fig. 1:**
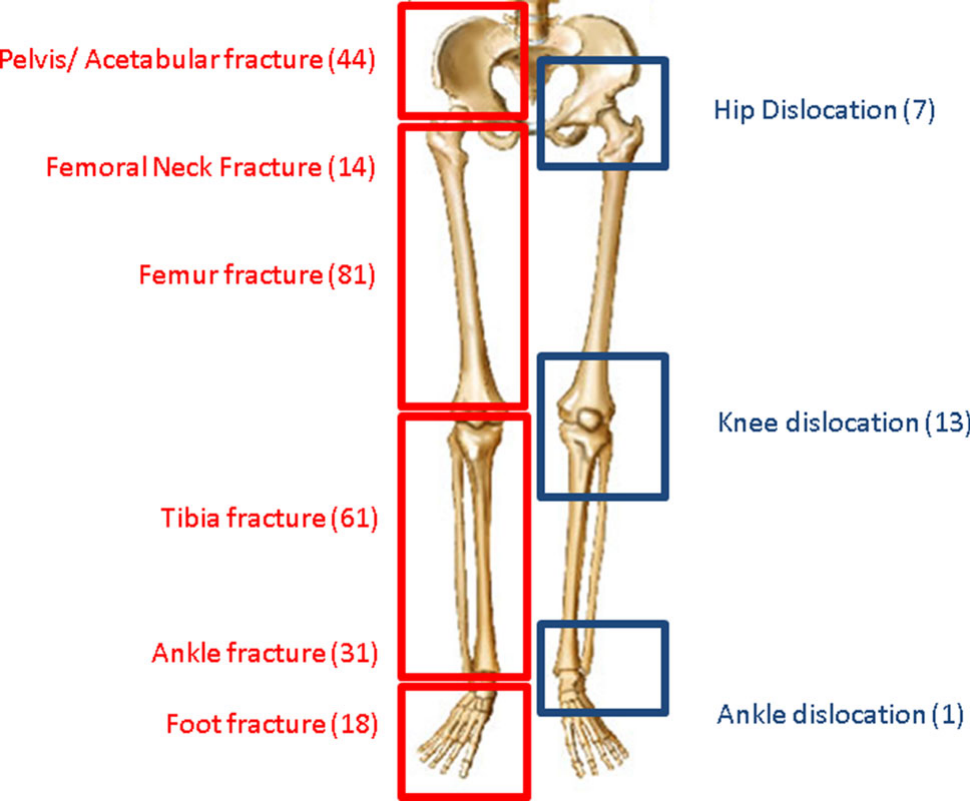
Distribution of lower extremity fractures (*left side, red*) and dislocations (*right side, blue*) associated with venous thromboembolism

One hundred and forty-two of the 167 patients sustained an isolated orthopaedic trauma injury, and 25 (15 %) patients were polytrauma patients with multiple diagnoses (range 2–4), to give a total of 270 lower extremity orthopaedic injuries. The injury distribution of fractures was as follows: pelvis/acetabulum (44), femoral neck (14), femoral shaft (81), tibia (61), and ankle/foot (49). The distribution of dislocations was hip (7), knee (13), and ankle (1). Figure 1 lists the injuries by type and anatomic distribution.

One hundred and twenty-one of the 167 (72 %) patients had at least one anticoagulant simultaneously coded with VTE and lower extremity orthopaedic trauma. One hundred and eleven (66 %) patients were treated with low-molecular-weight heparin, 28 (17 %) were treated with warfarin, and 11 (7 %) were treated with aspirin. Twenty-four patients (14 %) had more than one anticoagulant coded during the encounter.

## Discussion

Venous thromboembolism events in children are rare. Known risk factors include placement of a central venous catheter, obesity, trauma, and infection [4, 5, 10]. Rates of incidence of VTE in hospitalized children are increasing over time [11], with up to 58 cases reported per 10,000 hospital admissions [12]. In the general pediatric trauma population, the published incidence rates of VTE range from 0.02 to 0.26 % [13–15].

Georgopoulos et al. [8] used the PHIS database to calculate the incidence of VTE events associated with elective pediatric orthopaedic surgery [8]. They found a total of 71 cases over a 6-year time period within approximately 143,000 admissions, which corresponded to an incidence of 0.015 %. Greenwald et al. [4] reviewed all cases of pelvic and femoral fractures at their institution, and found a total of three cases of DVT during a 20-year time period, and reported a prevalence of 0.17 %. Our reported incidence of VTE after lower extremity orthopaedic trauma of 0.058 % is comparable with these two previous studies, reflecting consistent trends in VTE events over time.

In our series, the most common location for a lower extremity injury associated with VTE was a femur/femoral neck fracture, accounting for 40 % (95/270) of all injuries. Other common locations included fractures of the tibia/ankle, and fractures of the pelvis and acetabulum. This finding supports previous work which found that pediatric patients with lower extremity fracture are at increased risk for VTE [10]. Sabharwal and coauthors [5] surveyed members of the Pediatric Orthopaedic Society of North America, reporting first a preliminary survey of clinical experience with VTE in pediatric orthopaedic patients. In their study, more than half of the respondents could recall at least one case of DVT during their career. In a follow-up survey, Sabharwal et al. [6] reported on 46 children who suffered a VTE event as result of either trauma or elective orthopedic surgery. Most VTE events were associated with lower extremity procedures, such as osteotomies and fixation of a long-bone fracture.

Treatment for VTE commonly includes anticoagulation with low-molecular-weight heparin (LMWH), warfarin, or aspirin. We found that almost 75 % of patients in our cohort had an anticoagulant simultaneously coded with VTE and orthopaedic injury. Most treated patients received LMWH, and others also received warfarin or aspirin. The national data in our series confirms previously published work from the PHIS database from 2001 to 2008 which found that use of LMWH (enoxaparin) has been increasing in pediatric trauma patients [14]. Furthermore, the majority of providers in Sabharwal et al.’s survey reported using LMWH to treat VTE in pediatric orthopaedic patients [9].

Several limitations exist in this study. While there is merit in using databases such as the PHIS to report on a large volume of patients, there are inherent weaknesses with the accuracy of this database. It is possible that our reported incidence is an underestimation of the true incidence of VTE associated with pediatric orthopedic trauma, as it is likely that not all cases of VTE were reported or correctly coded. However, despite these limitations we believe that important conclusions can be drawn from this study.

In conclusion we describe the presentation and incidence of pediatric VTE across the United States associated with lower extremity orthopaedic trauma. The incidence of VTE after pediatric orthopaedic trauma is low and appears to be commonly associated with fractures of the femur, tibia, and pelvis/acetabulum. From our analysis, adolescents (≥12 years) appear to experience a higher incidence of VTE than infants and children. Clinicians caring for children and adolescents after orthopaedic lower extremity trauma should be aware of the risk of VTE.
